# Dosimetric parameter determination of a carbon-nanotube based miniature x-ray tube for HDR brachytherapy

**DOI:** 10.3389/fonc.2025.1628318

**Published:** 2025-11-19

**Authors:** Sang-Won Kang, Jin-Beom Chung, Keun-Yong Eom, Changhoon Song, In-Ah Kim, Jae-Sung Kim, Jeong-Woo Lee, Woong Cho

**Affiliations:** 1 Department of Radiation Oncology, Seoul National University Bundang Hospital, Seongnam, Republic of Korea; 2 Department of Radiation Oncology, Kunkuk University Medical Center, Seoul, Republic of Korea; 3 Department of Radiation Oncology, Seoul National University Boramae Medical Center, Seoul, Republic of Korea

**Keywords:** dosimetric parameters, HDR brachytherapy, vacuum-sealed miniature X-ray tube (mXT), AAPM task group (TG) 43, film dosimetry

## Abstract

**Purpose:**

This study aims to determine key dosimetric parameters of a vacuum-sealed miniature X-ray tube (mXT) equipped with a carbon nanotube field emitter for application in HDR brachytherapy.

**Methods:**

Dosimetric parameters, including dose-rate constant, radial dose, and anisotropic function, were assessed 1 cm below the mXT employing EBT3 film and a custom-manufactured acrylonitrile butadiene styrene (ABS) phantom. The dose-rate constant and radial-dose functions were measured following the standard polar angles and radial distances prescribed by the AAPM TG-43 protocol. However, anisotropic function measurements were selectively conducted due to the directional dependence of Gafchromic EBT3 film when placed coplanar to the X-ray source. To minimize this effect, films were positioned 1 cm below the mXT, which restricted the measurable angular range. These parameters were also computed in both a virtual ABS and water phantom using the MCNP6.1 code. Correlation factors for different materials were obtained to adjust measured parameters in the ABS phantom to those in water, based on the calculated depth–dose curve. The dosimetric parameters were then determined by comparing the measured and calculated values.

**Results:**

The dose-rate constant was determined to be 1344.14 cGy·h^-1^·μA^-1^. Radial-dose functions were 0.49, 0.33, 0.22, and 0.15 at radial distances of 2.0, 3.0, 4.0, and 5.0 cm, respectively. The difference between measured and calculated radial-dose functions in water remained within 0.10, averaging 0.05. Anisotropic functions exhibited an increase with the radial distance, approaching 0° angle. Azimuthal angular dependence was deemed acceptable.

**Conclusion:**

This study successfully acquired both measured and calculated parameters for the newly developed mXT. The findings affirm that the dosimetric parameters of the mXT are within acceptable limits for clinical HDR brachytherapy applications.

## 1 Introduction

High-dose-rate (HDR) brachytherapy is a well-established modality for cancer treatment, offering precise dose delivery while minimizing exposure to surrounding tissues (1, 2). Despite these benefits, traditional radiation sources such as ^192^Ir and ^60^Co present certain drawbacks, involving ongoing costs and potential hazards due to their specific half-lives (e.g., 73.8 days for ^192^Ir and 5.26 years for ^60^Co) (3, 4). Uncontrolled radioactive decay during treatment poses risks of unnecessary irradiation for both staff and patients.

To address these challenges, electronic brachytherapy (eBT) systems such as the Xoft Axxent (Xoft, Inc., Sunnyvale, CA) (5, 6) and the Intrabeam™ (Carl Zeiss Surgical, Oberkochen, Germany) (7, 8) have been introduced as alternatives to conventional radionuclide sources like Ir−192. These eBT systems offer distinct advantages, including on−demand X−ray generation, elimination of isotope decay and storage requirements, and simplified shielding due to their low−energy photon spectra. However, they also present notable limitations. For example, the Xoft Axxent source operates at 50 kVp with a relatively short operational lifetime (typically < 2.5 hours) and limited output stability over extended use. Moreover, conventional thermionic cathodes used in these eBT tubes require continuous filament heating, which generates significant internal heat, accelerating cathode degradation and reducing source stability. To manage this heat, active cooling systems are often required (4, 9), adding complexity and potentially limiting applicator compatibility. Similarly, the Zeiss Intrabeam system is primarily optimized for intraoperative radiotherapy and is less versatile for HDR brachytherapy applications requiring various applicator geometries. These limitations have hindered broader clinical adoption of current eBT technologies for routine HDR brachytherapy.

Our collaborative research institute, the Korea Advanced Institute of Science and Technology (KAIST), has developed a vacuum-sealed miniature X-ray tube (mXT) utilizing a novel carbon nanotube (CNT) field emitter (10). Unlike thermionic electron sources, CNT field emitters generate electrons through quantum tunneling, enabling high current output and enhanced operational stability (11–13). The CNT field emitter boasts advantages such as (1) the ability to control simple and easy pulse operation (14, 15), (2) the generation of high current for electron/X-ray microscopy devices (16, 17), and (3) independence from a cooling system (18). Consequently, the mXT exhibits a higher emission current, superior stability, and an extended lifetime (>100 h) compared to other commercially available electron sources. Compared with Ir-192, the mXT allows for electrical control of radiation emission, enhancing patient safety and treatment flexibility. Additionally, in contrast to the Xoft Axxent system, the mXT demonstrates a higher emission current, greater operational stability, and an extended lifetime exceeding 100 hours—features that contribute to improved clinical practicality and source longevity.

As reported in a previous study by Heo et al. (10), the mXT operates at voltages up to 70 kVp. During testing, the measured emission current at the CNT cathode was nearly identical to the current collected at the target anode under vacuum conditions, indicating efficient and stable electron transport within the sealed structure. Based on these operating characteristics, the mXT demonstrated an air-kerma strength (S_K_) of 108.1 Gy·cm²·min^-1^ at an operating voltage and current of 50 kVp and 252 μA—approximately 15 times higher than the typical air-kerma strength of a 10-Ci ^192^Ir HDR source (~7 Gy·cm²·min^-1^). Additionally, the earlier study described the spatial dose distribution as relatively uniform based on measurements in air. However, no quantitative TG-43 dosimetric parameters were determined at that time. That study also explored the mXT’s application in dental radiography and superficial electron brachytherapy for skin cancer, highlighting its potential as an alternative to commercialized electronic sources for HDR brachytherapy.

To facilitate the clinical application of the mXT in HDR brachytherapy, determination of its dosimetric parameters becomes imperative. These parameters, crucial for calculating the prescribed dose, align with the guidelines outlined by task group (TG) 43 of the American Association of Physicists in Medicine (AAPM) (19). Typically established through a comparative analysis of measured data with dosimeter devices and calculated data using Monte Carlo (MC) simulation (20–23), the dosimetric parameters of our mXT have yet to be determined. While previous studies have addressed certain dosimetric properties, such as air-kerma strength and dose distribution in the air (10), this study aims to comprehensively determine these parameters according to the TG-43 protocol for the application of the mXT in HDR brachytherapy.

## 2 Materials and methods

### 2.1 Carbon-nanotube field emitter based miniature X-ray tube

**Figure 1:**
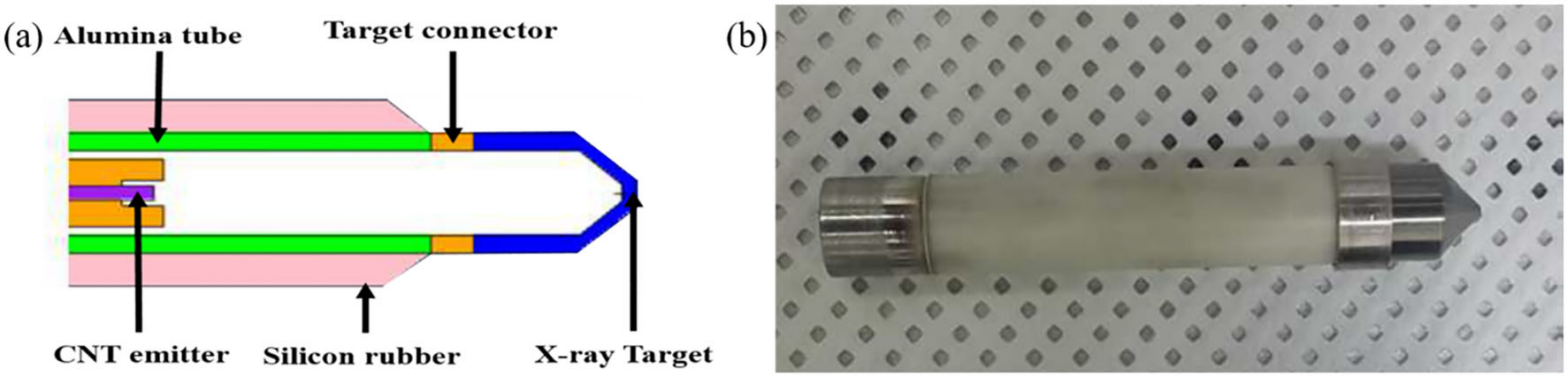
**(a)** Schematic of a vacuum-sealed miniature X-ray tube (mXT) based on a novel carbon nanotube field emitter and **(b)** manufactured mXT.

**Figure 1** depicts a schematic of the mXT, which has an outer diameter of 7 mm and an overall length of 47 mm. While the schematic in **Figure 1** was not intended to provide full geometric modeling detail, key dimensions were referenced in the simulation model. Specifically, we now state that the total tube length was modeled as 47 mm, the outer diameter was 7 mm, the conical target region was approximated with a slant length of 4.2 mm, and the exit window at the tip was set to 1 mm in thickness. Although the exact thickness of the tungsten coating was not modeled separately, these structural details were incorporated into the Monte Carlo geometry to reflect the physical construction of the mXT as closely as possible. The diode structure comprises a CNT field emitter serving as the cold cathode and a tungsten-coated beryllium conical transmission target functioning as the anode. The electron optics were optimized using EGN2 simulation to ensure efficient focusing of the emitted electrons onto the target while avoiding beam interception on the surrounding ceramic insulator, which could otherwise lead to high-voltage breakdown. To further improve dielectric stability, the outer surface of the target assembly was coated with a 2-mm silicone rubber layer.

The cathode, target, and ceramic tube were hermetically sealed within a vacuum envelope using Kovar and beryllium components with matched thermal expansion coefficients. A non−evaporable getter was incorporated to maintain high vacuum quality during long−term operation, as described by Heo et al (10). These design features enable stable electron emission up to 70 kVp with minimal heat generation because the CNT cathode operates via cold field emission rather than thermionic heating. This cold−emission mechanism significantly reduces power consumption and thermal stress, which improves the operational lifetime compared with conventional thermionic miniature x−ray tubes.

### 2.2 Dosimetric parameters of the mXT

Dosimetric parameters, including air-kerma strength, dose-rate constant, geometric function, and radial-dose/anisotropic functions, are typically determined according to TG-43 guidelines. However, in this study, only the dose-rate constant, radial dose function, and anisotropy function were experimentally measured. The S_K_ value of 108.1 Gy·cm²·min^-1^ was adopted from a previous study (9), where it was calculated using MC simulation under free-air conditions. Due to the unavailability of dedicated free-air ionization chambers (FAICs) for electronic X-ray sources in Korea, direct experimental measurement of S_K_ was not feasible. Thus, the simulation-based value was used as a reference for the calculation of the dose-rate constant in this study.

**Figure 2:**
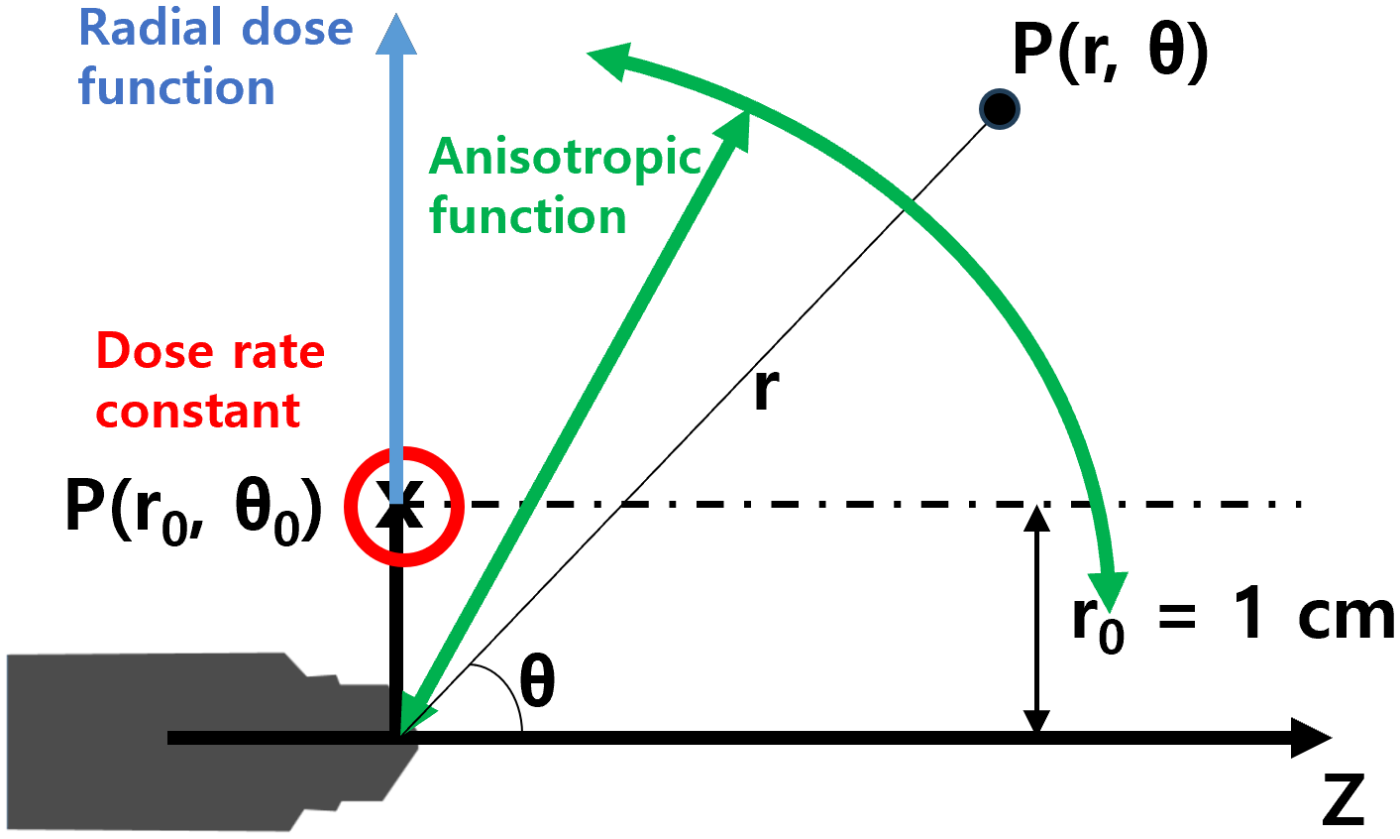
Coordinate system for dosimetric parameters of the mXT, such as the dose-rate constant, radial dose function, and anisotropy function.

**Figure 2** shows the coordinate system used for the dose-rate constant, radial dose, and anisotropic function. The dose-rate constant (Λ) is defined as the dose per S_K_ at the reference position, P(*r_0_, θ_0_*), situated at a radial distance (*r*) of 1 cm from the center of the mXT (reference radial distance, *r_0_* = 1 cm), with a standard polar angle of 90° (*θ_0_*). The value of Λ is derived from Equation 1 as follows:

(1) $$\Lambda =\frac{D(r_{0},\theta_{0})}{S_{K}}.$$

In contrast to conventional radiation sources, the determination of Λ for the mXT is based on the current, aligning with the approach used for other electronic brachytherapy sources (6). Therefore, in this study, the unit of Λ is considered to be cGy·h^-1^·μA^-1^.

Geometric functions are categorized into point- (G_P_) and line-source (G_L_) models. Typically, TG-43 recommends the use of G_L_ models due to their greater accuracy in dose calculations compared to G_P_ models. However, the mXT, deviating from conventional radiation sources with specific volumetric shapes, cannot employ conventional geometric functions. Therefore, the geometric functions for the mXT are determined using virtual point-source models, represented by G_P_(*r*) = 1/*r*^2^ throughout this study.

The radial-dose function (g(*r*)) signifies the dose fall-off on the transverse plane due to photon scattering and attenuation. g(*r*) is normalized to the dose at P(*r_0_, θ_0_*) (D(*r_0_, θ_0_*)) and can be calculated using G_P_(*r, θ*) as defined in Equation (2):

(2) $$g(r)=\frac{D(r,\theta_{0})}{D(r_{0},\theta_{0})}\cdot \frac{G_{P}(r_{0},\theta_{0})}{G_{P}(r,\theta_{0})}$$

The anisotropy function (F(*r, θ*)) is defined as the variation in dose with respect to the polar angle, relative to the transverse plane. F(*r, θ*) reflects the irregular distribution of dose from the mXT, attributed to the energy dependence on the direction and shape of the tube. Normalized to the dose obtained at the same *r* and at *θ_0_*, F(r, θ) is recalculated using G_P_(*r, θ*) as defined in Equation (3):

(3) $$F(r,\;\theta )=\frac{D(r,\theta )}{D(r,\theta_{0})}\cdot \frac{G_{P}(r,\theta_{0})}{G_{P}(r,\theta )}.$$

### 2.3 Measurement of dosimetric parameters

**Figure 3:**
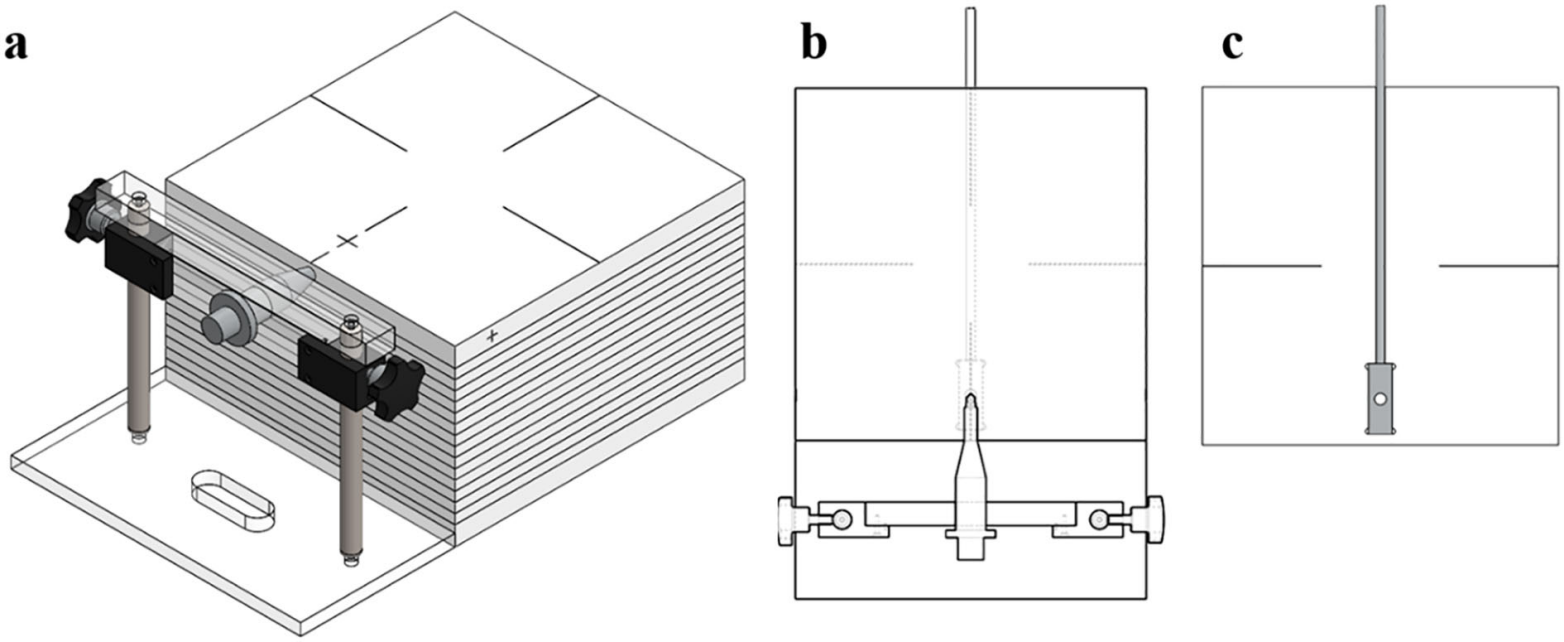
**(a)** Mimic diagram of the completed quality assurance phantom for the mXT, **(b)** source inserter slabs, and **(c)** XR multidetector inserter.

For the measurement of the dosimetric parameters, a dedicated ABS phantom (30 × 30 × 20 cm³, density = 1.04 g/cm³) was fabricated, as illustrated in **Figure 3**. The phantom consisted of a source holder slab and detector positioning slots to accommodate the mXT and Gafchromic EBT3 films (ISP, batch no. 04181701) as well as an XR multidetector (MagicMax Universal XR, IBA Dosimetry, Germany).

**Figure 4:**
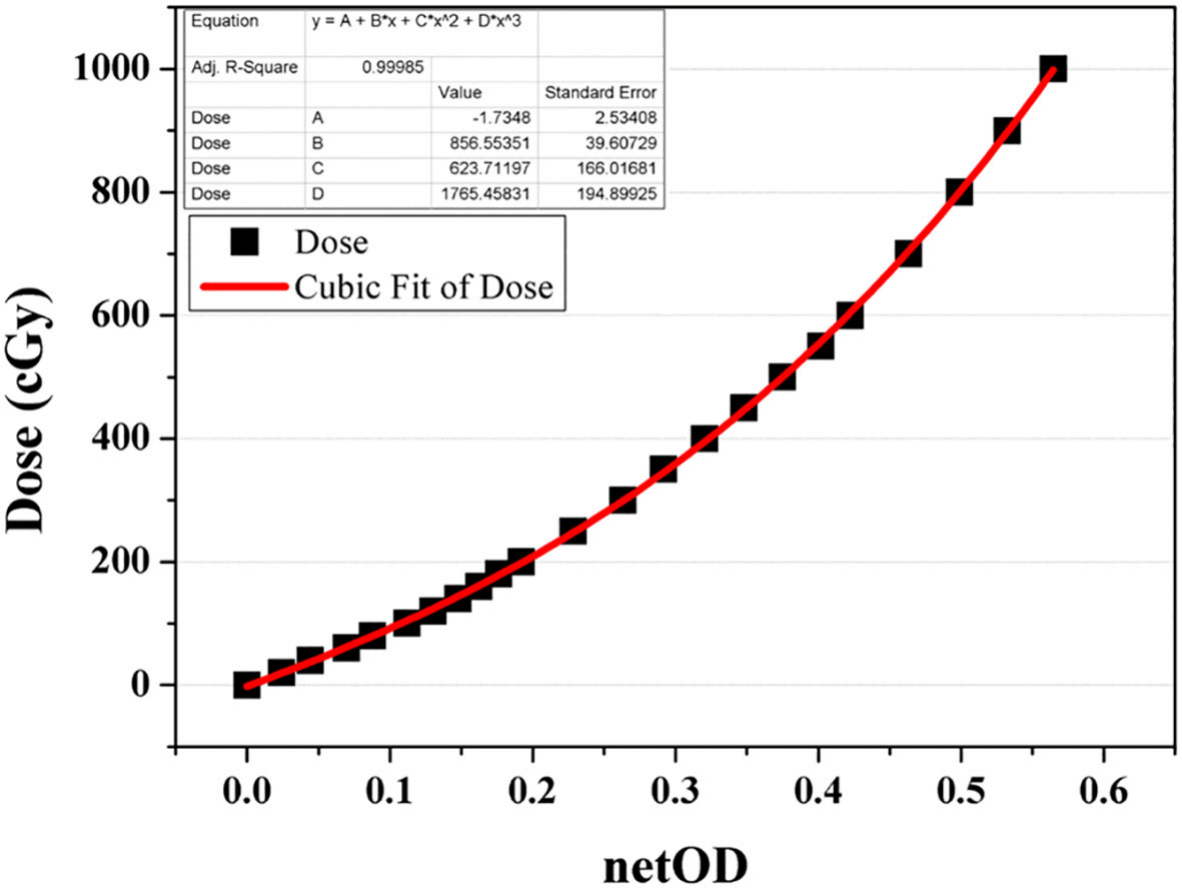
Net optical-density (netOD) curve acquired as a function of dose from a 6-MV beam for EBT3 film calibration.

The dosimetric parameters were measured using the Gafchromic EBT3 film (International Specialty Products, ISP, Wayne, NJ, batch number: 04181701) and an XR multidetector (MagicMax Universal XR Multidetector, IBA Dosimetry GM bH, Germany). Film calibration involved the use of a 6-MV external photon beam from VitalBeam™ (Varian Medical Systems, Palo Alto, CA, USA) to obtain the net optical-density (netOD) curve, as depicted in **Figure 4**. Following a 24 h period, all irradiated films were scanned at a resolution of 75 dpi using a flatbed scanner (Epson Expression 11000 XL, Epson America Inc., Long Beach, CA). A calibration curve relating netOD to dose was constructed and fitted using a third-order polynomial, as recommended by AAPM TG-55 and relevant literature. This calibration curve was then applied to convert experimental film readings to absorbed dose.

EBT3 films were positioned 1 cm below the mXT within the ABS phantom for TG-43 parameter measurements. While TG-43 recommends measuring parameters coplanar with the source in water, our measurements were performed in ABS. To convert these data to water-equivalent values, Conversion factors for the materials (CF_M_) were derived by comparing MC–calculated depth-dose curves in ABS and water phantoms. After material conversion, the parameters were repositioned virtually to the coplanar TG-43 geometry.

Λ and g(*r*) were determined using the EBT3 film at the P(*r_0_, θ_0_*) position, with r ranging from 1.0 to 5.0 cm, respectively. Additionally, g(*r*) was measured using an XR multidetector at *r* from 1.0 to 5.0 cm in 1-cm increments, providing secondary verification of the film measurements. The value of F(*r, θ*) was measured with constraints on *r* based on the polar angle due to specific conditions related to the position for film dosimetry. F(*r, θ*) with polar angles below 20°could not be measured. To ascertain the azimuthal dependence of the mXT, doses were measured using an XR multidetector at a reference depth (1 cm), with the mXT rotated at 30° intervals from 0° to 180°.

### 2.4 Monte Carlo simulation of the mXT

The Monte Carlo N-particle version 6.1 (MCNP6.1) radiation transport code system (Los Alamos National Laboratory) was employed to calculate the dosimetric parameters of the mXT. The simulation modeled both ABS and water phantoms with dimensions of 30 × 30 × 20 cm³ to replicate the experimental setup. The ABS phantom was modeled in MCNP6.1 with a density of 1.04 g/cm³ and an elemental composition as 85.63% carbon, 7.13% hydrogen, and 7.24% nitrogen. The water phantom was modeled using standard ICRU-4 elemental composition (11.19% hydrogen, 88.81% oxygen) and a density of 1.0 g/cm³. The scoring voxel size was set to 1 × 1 × 1 mm³ to balance spatial resolution and statistical efficiency. Each simulation used 8 × 10^9^ particle histories, ensuring reliable dose estimation with low relative error. The cutoff energies were 1 eV for photons and 10 eV for electrons (24). MCNP6.1 has the capability to implement a comprehensive element-specific relaxation process utilizing the EPRDATA12 photo/electro-atomic cross-section library (25, 26). The global mean relative error across the entire scoring volume was calculated as 0.07%, which was obtained by applying dose weighted averaging over all scored voxels. These results confirm the reliability of the MC-derived parameters for subsequent comparison with measurements.

The uncertainties from film measurements were primarily evaluated to validate the consistency between ABS phantom measurements and MC simulations. These film-based uncertainties were not directly propagated into the final TG-43 parameters, which were instead derived from MC calculations. For MC–based TG-43 parameters, the propagated uncertainties included statistical tally errors, voxelization effects, and medium correction factors combined in quadrature, following the TG-138 recommendations.

Conversion factors for the materials (CF_M_) were introduced to convert doses measured in the ABS phantom into their water-equivalent values, in accordance with the TG-43 formalism. These factors were applied both to XR multidetector measurements and to doses measured with EBT3 film in ABS in order to enable direct comparison with water-based dosimetric parameters. The CF_M_ values were determined through MC simulations by calculating depth-dose curves under identical geometric and beam conditions for ABS and water phantoms. A distance-dependent conversion function was obtained by fitting the ratio of the two curves. This function was then applied to the ABS-based MC doses, and the converted results were compared with the directly calculated doses in water. The residual deviation between these two datasets was quantified using the root-mean-square error (RMSE), which was defined as the CF_M_ uncertainty.

## 3 Results

### 3.1 Material correlation factor and dose-rate constants

**Figure 5:**
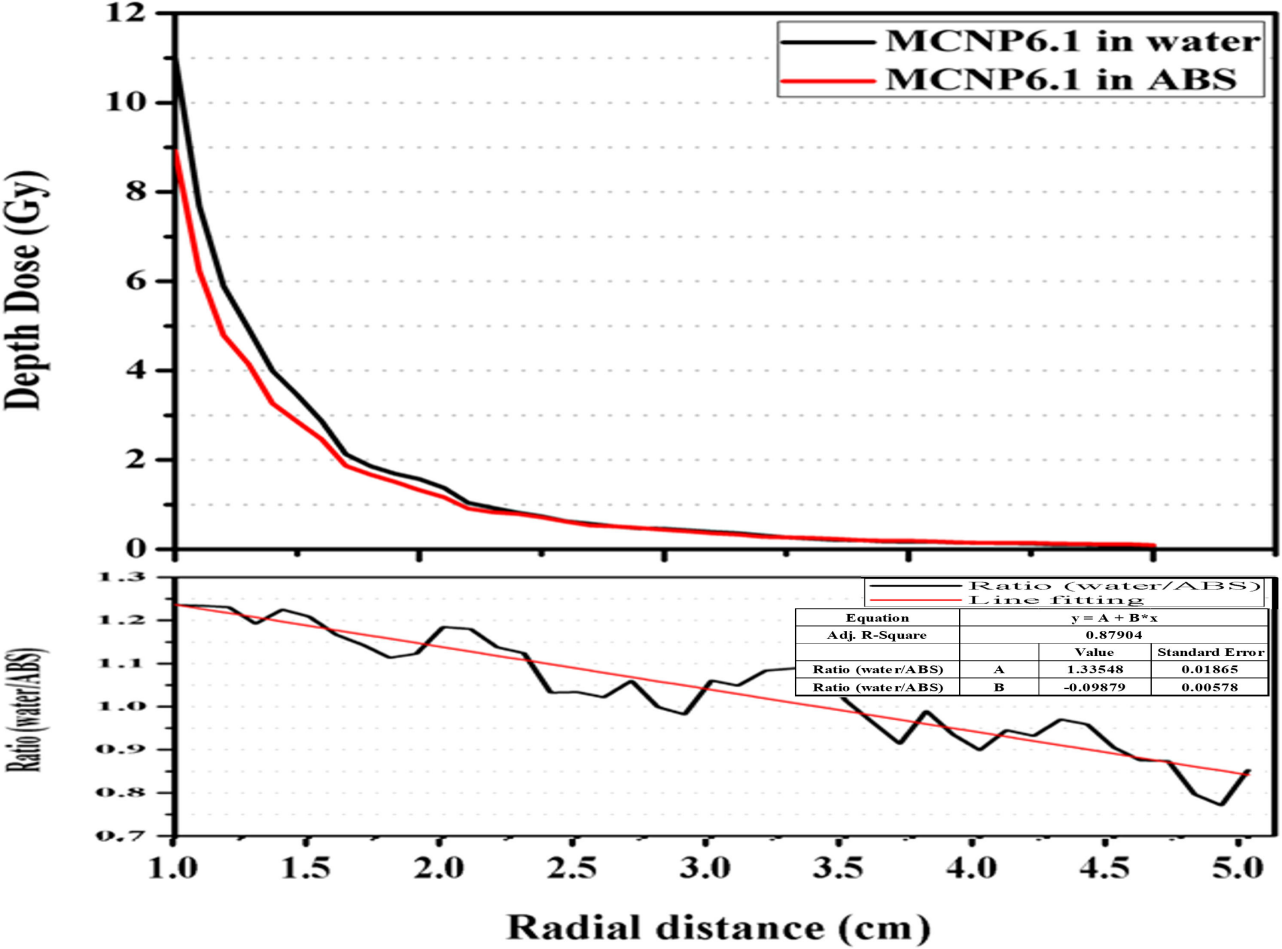
Calculated depth–dose curves by MCNP6.1 in virtual water and ABS phantom. The correlation factor defined as the ratio of the calculated depth–dose curve between both materials with respect to the radial distance.

As depicted in **Figure 5**, the CF_M_ were computed by establishing the ratio between the depth–dose curves of the mXT within the ABS and water phantoms. At the P(*r_0_, θ_0_*) position, the CF_M_ was 1.23. With an increase in the radial distance (*r*), the CF_M_ decreased, reaching 0.85 at an *r* of 5.0 cm.

In our experimental setup, the measured value of Λ in the ABS phantom (_ABS_Λ) was 1104.28 cGy·h^-1^·μA^-1^. This _ABS_Λ value was converted to the corresponding value in water (_water_Λ), equaling 1344.14 cGy·h^1^·μA^-1^, utilizing CF_M_. The standard deviation and maximum value of _water_Λ were 0.29 and 1345.64 cGy·h^-1^·μA^-1^, respectively.

### 3.2 Radial-dose function

**Table 1** presents the calculated and measured radial-dose functions, ^ABS/water^*g(r*)^MC/EBT^, where ABS/water denotes the phantom material and MC/EBT indicates whether the parameters are calculated by MCNP6.1 or measured using the EBT3 film, respectively.

**Table 1:** Calculated and measured radial-dose function (*g(r)*) data for the miniature X-ray tube (mXT) and comparison between the measured and calculated *g(r)*.

Radial distance, *r* (cm)	^ABS^g(*r*)^MC*^	^water^g(*r*)^MC^	^ABS^g(*r*)^EBT^	^water^g(*r*)^EBT^	^water^g(*r*)^MC^/ ^ABS^g(*r*)^MC^	^ABS^g(*r*)^MC^ - ^ABS^g(*r*)^EBT^	^water^g(*r*)^MC^ - ^water^g(*r*)^EBT^
1.0	1.00	1.00	1.00	1.00	1.00		
1.1	0.85	0.84	0.93	0.93	0.99	-0.08	-0.09
1.2	0.77	0.77	0.85	0.84	1.00	-0.08	-0.07
1.4	0.72	0.71	0.73	0.70	0.99	-0.01	0.01
1.6	0.71	0.67	0.60	0.57	0.94	0.11	0.10
1.8	0.61	0.55	0.53	0.50	0.90	0.08	0.05
2.0	0.60	0.57	0.51	0.47	0.95	0.09	0.10
2.2	0.50	0.46	0.45	0.41	0.92	0.05	0.05
2.4	0.51	0.43	0.43	0.38	0.84	0.08	0.05
2.6	0.47	0.39	0.38	0.33	0.83	0.09	0.06
2.8	0.45	0.37	0.37	0.31	0.82	0.08	0.06
3.0	0.44	0.38	0.34	0.29	0.86	0.10	0.09
3.2	0.41	0.36	0.34	0.28	0.88	0.07	0.08
3.4	0.37	0.33	0.31	0.25	0.89	0.06	0.08
3.6	0.37	0.29	0.28	0.22	0.78	0.09	0.07
3.8	0.33	0.26	0.26	0.20	0.79	0.07	0.06
4.0	0.34	0.25	0.23	0.18	0.74	0.11	0.07
4.2	0.31	0.24	0.21	0.16	0.77	0.10	0.08
4.4	0.31	0.24	0.22	0.16	0.77	0.09	0.08
4.6	0.30	0.21	0.21	0.15	0.70	0.09	0.06
4.8	0.30	0.19	0.18	0.12	0.63	0.12	0.07
5.0	0.26	0.18	0.16	0.11	0.69	0.10	0.07
				Mean	0.85	0.07	0.05
				SD^**^	0.10	0.05	0.05

^*water/ABS,^g(*r*) ^MC/EBT^: The ABS/water means materials of the calculated or measured QA phantom, and the MC/EBT means to calculate using MCNP6.1 and measure by EBT3 film, respectively.

**SD, standard deviation.

The overall ^water^*g(r)*^MC^ values were lower than ^ABS^*g(r)*^MC^, and this difference increased with an increase in *r*. Consequently, all ratios between ^water^*g(r)*^MC^ and ^ABS^*g(r)*^MC^ were below 1.0, decreasing as r increased. The average and standard deviation (SD) of the ratio were 0.85 and 0.10, respectively.

Differences between ^ABS^*g(r)*^MC^ and ^ABS^*g(r)*^EBT^ were 0.09, 0.10, 0.11, and 0.10 at r values of 2.0, 3.0, 4.0, and 5.0 cm, respectively. The maximum difference was 0.12 at an r of 4.8 cm. The average and SD of the differences were 0.06 and 0.06, respectively.

After correction for water, overall ^water^*g(r)*^EBT^ and differences between ^water^*g(r)*^MC^ and ^water^*g(r)*^EBT^ decreased compared with those before correction. The maximum absolute difference between the MC calculated and measured radial-dose functions was 0.10. The average and standard deviation of the absolute differences were 0.05 and 0.05, respectively. These values are summarized in **Table 1**. Measured dosimetric parameters are reported with standard deviations to reflect measurement uncertainty.

The *g(r)* values measured by the XR multidetector in the ABS phantom (^ABS^*g(r)*^XR^) were 0.54, 0.33, 0.25, and 0.20 at r values of 2.0, 3.0, 4.0, and 5.0 cm, respectively. Differences between ^ABS^*g(r)*^XR^ and ^ABS^*g(r)*^EBT^ were 0.03, -0.01, 0.02, and 0.04 at the corresponding r values, respectively. **Table 1**. Calculated and measured radial-dose function (g(*r*)) data for the miniature X-ray tube (mXT), and comparison between the measured and calculated g(*r*).

### 3.3 Anisotropic function

**Figure 6:**
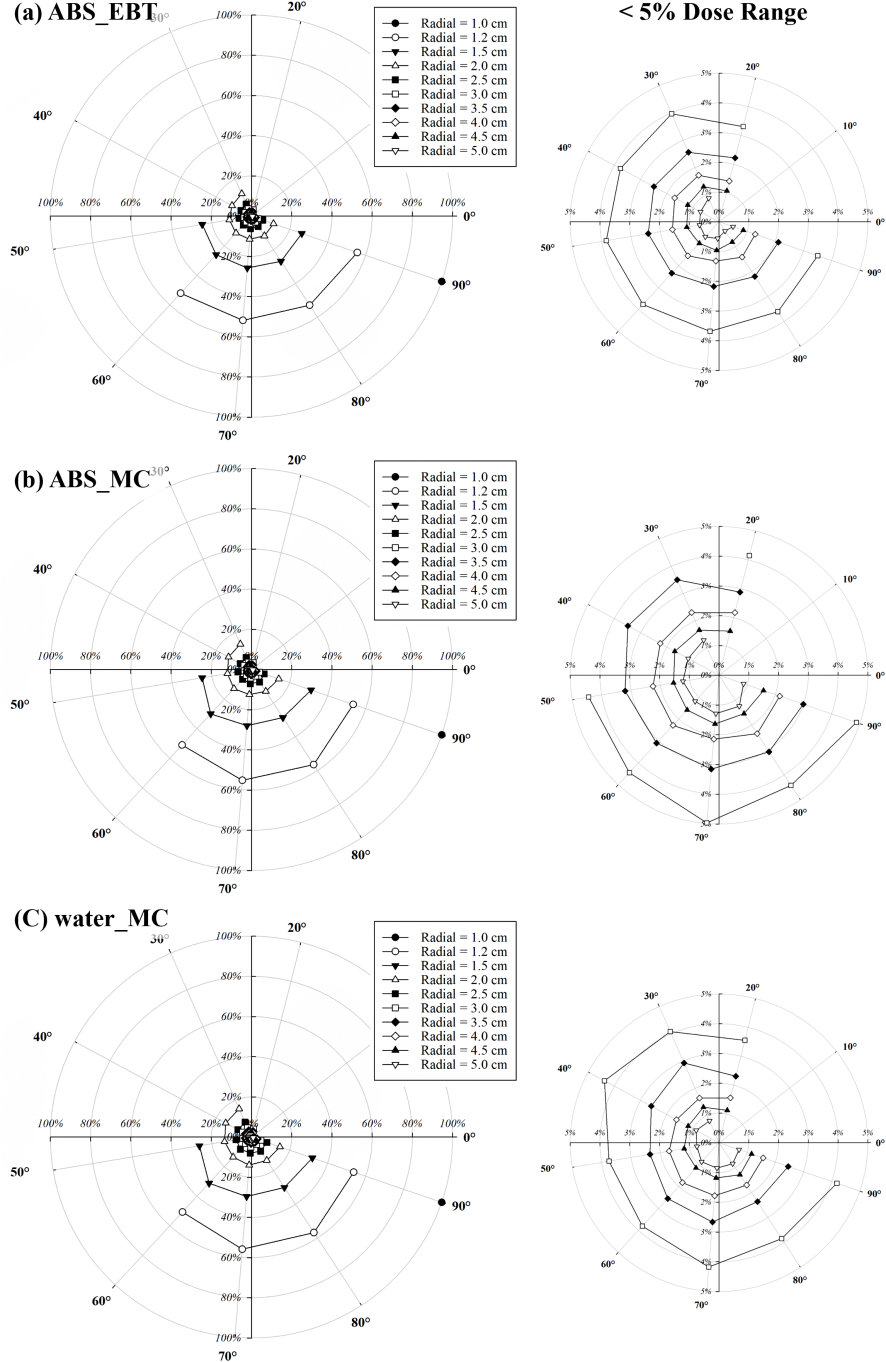
Angular and radial dose distributions normalized to 100% at the reference position of 90° and 1 cm from the source. Regions with relative doses below 5% are magnified for clarity. **(a)** shows relative doses measured in the ABS phantom using EBT3 film, while **(b, c)** present relative doses calculated via Monte Carlo simulations in the ABS and water phantoms, respectively.

**Figure 6** shows the relative angular dose distributions normalized to 100% at the reference position of 90° and 1 cm from the source, for radial distances ranging from 1 cm to 5 cm. Within the forward and lateral angular range (20°–90°), the relative dose decreased gradually as the polar angle deviated from 90°. The most pronounced angular dependence was observed near the source (r = 1 cm), where the relative dose dropped by more than 20% at 20°compared with the lateral reference. As the radial distance increased to 5 cm, the angular variation became less significant, indicating a smoother dose distribution in the far field.

**Table 2** summarizes the quantitative values of F(*r, θ*) derived from the same angular dose measurements presented in **Figure 6**, along with the ratio between ^water^F(*r, θ*)^EBT^ and ^water^F(*r, θ*)^MC^. The averages for the same *r* of ^water^F(*r, θ*)^EBT^ and ^water^F(*r, θ*)^MC^ were below 1.0 for *r* values up to 1.6 cm. However, the overall averages exceeded 1.0 when *r* was greater than 1.6 cm. The maximum averages of ^water^F(*r, θ*)^EBT^ and ^water^F(*r, θ*)^MC^ were 1.19 and 1.16 at 80° and 90°, respectively. As the angle approached 90°, the averages for the same angle of ^water^F(*r, θ*)^EBT^ and ^water^F(*r, θ*)MC tended to approach 1.0. The SD of ^water^F(*r, θ*)^EBT^ and ^water^F(*r, θ*)MC ranged from 0.01 to 0.11.

**Table 2:** Anisotropic function (F(*r*, θ)) data for the miniature X-ray tube (mXT) and the ratio between calculated and measured F(r, θ).

Anisotropic Function	*θ* (Degrees)	Radial distance, *r* (cm)																		Mean	SD
		1	1.1	1.2	1.4	1.6	1.8	2	2.2	2.4	2.6	2.8	3	3.2	3.4	3.6	3.8	4	4.2		
^water^F(*r, θ*)^EBT*^	20											1.02	0.94	0.98	1.08	1.02	1.07	1.10	1.21	1.05	0.08
	30							1.05	1.10	1.10	1.16	1.20	1.13	1.19	1.32	1.23	1.21	1.33	1.28	1.19	0.09
	40					0.94	0.97	0.96	1.03	1.00	1.12	1.14	1.07	1.16	1.19	1.22	1.18	1.32	1.30	1.11	0.12
	50				0.92	1.01	0.99	0.97	1.04	1.04	1.09	1.16	1.10	1.07	1.18	1.14	1.20	1.24	1.31	1.10	0.10
	60			0.94	0.93	1.01	1.00	0.99	1.05	1.02	1.05	1.08	1.08	1.06	1.11	1.12	1.11	1.21	1.15	1.06	0.07
	70		0.93	0.93	0.98	0.99	1.01	1.00	1.02	1.00	1.05	1.11	1.05	1.04	1.05	1.01	1.02	1.03	1.01	1.01	0.04
	80		0.97	0.95	0.99	0.98	1.01	1.01	1.01	1.01	1.06	1.09	1.03	1.00	1.11	0.98	0.98	1.10	1.13	1.02	0.05
^water^F(*r, θ*)^MC^	**Mean**		0.95	0.94	0.96	0.99	1.00	1.00	1.04	1.03	1.09	1.11	1.06	1.07	1.15	1.10	1.11	1.19	1.20		
	SD**		0.02	0.01	0.03	0.03	0.01	0.03	0.03	0.03	0.04	0.05	0.06	0.07	0.08	0.09	0.08	0.11	0.10		
	0	1.10	1.11	1.13	1.15	1.16	1.17	1.15	1.17	1.20	1.15	1.15	1.14	1.16	1.17	1.23	1.24	1.18	1.11	1.16	0.04
	10	1.03	1.05	1.01	1.05	1.09	1.04	1.05	1.05	1.09	1.07	1.07	1.03	1.08	1.13	1.18	1.18	1.22	1.12	1.09	0.06
	20	0.86	0.90	0.87	0.94	0.93	1.06	1.07	1.05	1.06	1.06	1.05	1.03	1.11	1.19	1.22	1.24	1.17	1.03	1.05	0.11
	30	0.97	0.89	0.95	0.96	1.10	1.07	1.04	1.01	1.12	1.08	1.06	1.12	1.15	1.14	1.18	1.19	1.20	1.13	1.08	0.09
	40	0.83	0.77	0.85	0.98	0.86	0.98	1.00	0.98	1.01	1.06	1.08	1.05	1.10	1.13	1.15	1.13	1.13	1.06	1.01	0.11
	50	0.83	0.76	0.83	0.97	0.86	0.99	0.98	0.97	0.98	1.02	1.01	0.99	1.02	1.00	1.08	1.08	1.09	1.04	0.97	0.09
	60	0.91	0.84	0.89	0.89	1.01	0.99	0.96	0.93	1.03	0.99	0.96	1.04	1.08	1.04	1.08	1.16	1.17	1.04	1.00	0.09
	70	0.89	0.83	0.89	0.86	0.86	0.99	0.98	0.95	0.96	0.92	0.93	0.95	1.01	1.07	1.15	1.17	1.14	1.03	0.98	0.10
	80	0.93	0.94	0.92	0.95	0.96	0.96	0.97	0.99	1.03	1.03	1.00	1.03	1.06	1.06	1.11	1.10	1.07	0.97	1.00	0.06
^water^F(*r, θ*)^EBT^ / ^water^F(*r, θ*)^MC^	**Mean**	0.93	0.90	0.93	0.97	0.98	1.03	1.02	1.01	1.05	1.04	1.03	1.04	1.09	1.10	1.15	1.17	1.15	1.06		
	**SD**	0.09	0.11	0.09	0.08	0.11	0.06	0.06	0.07	0.07	0.06	0.06	0.06	0.05	0.06	0.05	0.05	0.05	0.05		
	20											0.97	0.92	0.88	0.91	0.84	0.86	0.94	1.17	0.94	0.10
	30							1.02	1.09	0.98	1.07	1.13	1.01	1.03	1.16	1.04	1.02	1.11	1.13	1.07	0.06
	40					1.09	0.99	0.96	1.05	0.99	1.06	1.06	1.02	1.06	1.05	1.06	1.05	1.17	1.23	1.06	0.07
	50				0.95	1.17	1.00	0.99	1.07	1.06	1.07	1.15	1.11	1.05	1.18	1.06	1.11	1.14	1.26	1.09	0.08
	60			1.05	1.05	1.00	1.01	1.02	1.13	1.00	1.06	1.13	1.03	0.99	1.07	1.04	0.95	1.03	1.10	1.04	0.05
	70		1.12	1.05	1.14	1.15	1.01	1.02	1.07	1.05	1.15	1.19	1.11	1.03	0.99	0.88	0.87	0.90	0.99	1.04	0.10
	80		1.04	1.03	1.04	1.02	1.05	1.04	1.02	0.98	1.03	1.09	1.00	0.94	1.04	0.88	0.89	1.03	1.17	1.02	0.07
	**Mean**		1.08	1.04	1.04	1.09	1.01	1.01	1.07	1.01	1.07	1.10	1.03	1.00	1.06	0.97	0.96	1.04	1.15		
	**SD**		0.03	0.01	0.06	0.06	0.02	0.02	0.03	0.03	0.03	0.06	0.06	0.06	0.08	0.09	0.08	0.09	0.08		

*waterF(*r*, θ) MC/EBT: MC/EBT is calculated using MCNP6.1 and measured based on the EBT3 film.

**SD, Standard deviation.

The maximum and minimum of the ratio were 1.26 (*r* = 4.2 cm, *θ* = 50°) and 0.84 (*r* = 3.6 cm, *θ* = 20°), respectively. The maximum average ratio for the same *r* was 1.15 at an *r* of 4.2 cm. Overall average ratios for the same *r* were higher than 1.0, except at r values of 3.6 and 3.8 cm. The SD of the ratio ranged from 0.01 to 0.10. In general, the SD of the ratio increased as *r* increased.

### 3.4 Azimuthal angular dependence

**Table 3** shows the azimuthal angular ratios at 30° intervals normalized to the dose at 0°for the mXT. The maximum ratio of azimuthal angular was 1.06 at 150°. In particular, the ratios for 90° and 180° were similar to that for 0˚.

**Table 3:** Azimuthal angular dependence of the miniature X-ray tube (mXT).

Azmothal angle	Relative
0 ˚	1.00
30 ˚	1.04
60 ˚	1.05
90 ˚	1.01
120 ˚	1.03
150 ˚	1.06
180 ˚	1.02

### 3.5 Uncertainty analysis

As shown in **Table 4**, the total uncertainty was computed using the quadrature sum of individual components, following the approach outlined in AAPM TG-138 (27) and other relevant reports (28–31). Type A uncertainty, reflecting random experimental variations, was assessed through repeated EBT3 film measurements. Type B uncertainties represent systematic effects associated with film dosimetry, geometry, and beam characteristics. In particular, the energy dependence of the EBT3 film, which dominates the total uncertainty, was not directly measured in this study but adopted from previously published investigations by Massillon-JL et al. (29) and Sutherland et al. (30), both of which evaluated EBT3 response under kilovoltage polyenergetic X-ray beams. The reported deviation in dose response under such conditions supports the conservative 11% uncertainty assigned in our analysis. Calibration uncertainty was derived from residuals in the dose–netOD fitting process using a 6 MV photon beam, and setup-related geometric uncertainty was estimated based on a ±1 mm tolerance, consistent with prior studies such as that of Bekerat et al. (31) Additionally, the medium correction factor was obtained through Monte Carlo simulations comparing dose deposition between ABS and water-equivalent materials under mXT conditions, accounting for spectral and compositional differences.

**Table 4:** Uncertainty analysis of the EBT3 measurement and Monte Carlo calculation.

EBT3 uncertainty			
Component	Type A		Type B
Repetitive measurements	0.04%		
Film calibration uncertainty using 6 MV photon beam of Linac (29)			0.50%
EBT3 measured uncertainty in low energy (30)			11.00%
Medium correction factor (CF_M_)			4.72%
Geometric uncertainty (31)			0.98%
Quadrature sum	0.04%		12.02%
Total uncertainty		12.06%	

Monte Carlo uncertainty			
Component	r = 1 cm		r = 3 cm
Statistics	1.32%		3.58%
geometric uncertainty	4.95%		2.49%
Quadrature sum	5.12%		4.36%

For the MCNP6.1 calculations, statistical uncertainty was extracted from the relative error values and averaged over scoring voxels within 1-mm radial regions at distances of 1.0 cm and 3.0 cm. Geometric uncertainty was evaluated by analyzing dose variation across voxels within each region and was quantified using a uniform distribution–based standard deviation approximation. Specifically, it was computed as (max–min)/(2 × mean × √3), where max and min refer to the highest and lowest dose values within the scoring region. While not formally prescribed in TG-138, this approach addresses spatial sensitivity to voxel alignment, which becomes particularly pronounced near the source. As a result, the statistical uncertainty at 1.0 cm was lower than at 3.0 cm due to higher particle fluence, while geometric uncertainty was greater at 1.0 cm due to stronger dose gradients. The overall combined MC uncertainties were 5.12% and 4.36% at 1.0 cm and 3.0 cm, respectively.

## 4 Discussion

The dosimetric parameters obtained from measurements were converted to values in water using CF_M_, as determined by comparing depth–dose curves in ABS and water (**Figure 5**). Unlike in our study, Gary (32) calculated correction factors for materials such as PMMA, solid water, and water by comparing radial-dose functions rather than depth–dose curves. To validate our CF_M_, we obtained ^water^g(*r*)^MC^/^ABS^g(*r*)^MC^ (**Table 1**), representing correction factors calculated following Gary’s method. The trend of ^water^g(*r*)^MC^/^ABS^g(*r*)^MC^ closely mirrored Gary’s correction factors. In a PMMA phantom, Gary’s correction factor at P(*r_0_, θ_0_*) for ^103^Pd was 0.737, while the inverse of our CF_M_ at the same position was 0.82. This discrepancy may be attributed to energy differences between ^103^Pd and mXT, as well as variations in phantom materials (ABS and PMMA). Consequently, we have demonstrated the relevance of our CF_M_ for material corrections.

The observed trend that ^ABS^g(*r*)^MC^ values were higher than those in water, particularly at greater distances, can be explained by the intrinsic differences in photon attenuation and the normalization scheme used in TG-43 formalism. The ABS, composed mainly of carbon and hydrogen, has a lower effective atomic number than water, leading to weaker attenuation of low-energy photons and consequently allowing more dose to be deposited at extended radial distances. Furthermore, because g(*r*) is defined relative to the dose at 1 cm, the steeper near-field dose in water causes normalized g(*r*) values to drop more sharply with increasing r. These combined effects can contribute to the increasing discrepancy observed between water and ABS g(*r*) values.

In g(*r*), differences between ^water^g(*r*)^MC^ and ^water^g(*r*)^EBT^ were lower than those between ^ABS^g(*r*)^MC^ and ^ABS^g(*r*)^EBT^. This is attributed to the EBT3 film being composed of a water-equivalent substance. Within the ABS phantom, the EBT3 film was defined as a heterogeneity slice by comparison with ABS. Although this slice thickness was sufficiently thin not to significantly affect measurements, the measured uncertainty might have increased relative to a homogeneous environment. However, these differences were minor, and this uncertainty was included in the CF_M_. Thus, the overall measured g(*r*) was deemed appropriate and acceptable when compared with calculated g(*r*).

F(*r, θ*) measurements were limited to specific ranges: angles greater than 20° and variable r based on the angle. This limitation arose from the specific conditions of EBT3 film measurement, where the film was positioned 1 cm below the mXT. This phenomenon arose due to the requirement that F(r, θ) could only be measured when the length of a exceeded 1.0 cm. The purpose of this measurement was to mitigate the directional dependence of the film. When the film was positioned coplanar to the mXT, the angle between the film and mXT was 90°. Typically, the film exhibited directional dependence at 90° (33). Consequently, we positioned the film 1 cm below the mXT to circumvent the directional dependence observed in the film.

**Figure 6** shows that angular dose dependence is most pronounced in the near field, where the steep dose gradients result in larger relative differences across polar angles. As the radial distance increases, the angular variation becomes less significant, leading to a smoother dose distribution in the far field. This behavior is consistent with the expected beam divergence and self-attenuation characteristics of a miniature X-ray tube. Clinically, this suggests that while angular positioning is critical in the immediate vicinity of the source, its influence on dose uniformity diminishes at greater distances, potentially simplifying treatment planning for larger target volumes.

Measured and calculated F(*r, θ*) (**Table 2**) exhibited a similar trend, increasing with *r* and approaching 0°. This behavior is attributed to the angular dependence of bremsstrahlung photon emission, which is inherently forward-directed due to the momentum transfer from high-energy electrons to the target material. As the radial distance increases, this directional bias becomes more pronounced in the dose distribution, resulting in an increased anisotropy function particularly near the 0° axis (34).

The azimuthal angular dependence presented in **Table 3** reflects the rotational symmetry of the mXT around its longitudinal axis. A slight azimuthal variation was observed in the near field, with dose differences measured within 6%. Although this variation is relatively small, it could potentially influence dose uniformity in certain clinical scenarios, particularly in complex treatment geometries or non-coplanar setups. To address this, the measured azimuthal variation will be incorporated into the development of a dedicated radiotherapy treatment planning (RTP) system for the mXT, allowing more accurate dose calculations that account for such angular dependencies. Furthermore, since the current mXT is a prototype, future manufactured units may exhibit slight structural or dosimetric deviations. Therefore, these parameters should be continuously measured and validated for each newly produced device to ensure long-term reliability and reproducibility of the source’s dosimetric characteristics.

Although the dose distribution of the mXT approximated a spherical shape due to its low energy (lower than 50 keV), the energy of the emitted photons in the direction of the electron path (*θ* = 0°) was higher than in other directions. Despite these trends, the ratios between F(*r, θ*) were mostly within an acceptable range, considering dosimetric uncertainty. Furthermore, the overall uncertainty remained within the ±10% range recommended by AAPM TG-56 (35) for brachytherapy dose delivery, supporting the dosimetric feasibility of the mXT for HDR applications.

In the uncertainty analysis, the measured uncertainty of the EBT3 component at low energy was the highest at 11.00%. While the film offers advantages such as high resolution, water equivalence, and the ability to measure 2-D dose distribution, it exhibits energy dependency in the low-energy region (<1.25 MeV). Consequently, the uncertainty of EBT3 for dosimetric parameter measurements increased to 12.06%. The uncertainty of CF_M_ was calculated as 4.72% to assess the accuracy between converted and calculated results using MCNP6.1. Comparing geometric and statistical uncertainties in MCNP6.1, we found that the statistical uncertainty within a radial distance of 1 cm was lower than at 3 cm, while geometric uncertainty at 1 cm was higher due to the steeper dose gradient near the source. It is worth noting that the relatively larger uncertainty observed in the EBT3 film measurements served mainly as a benchmark to assess the reliability of MC-derived dose distributions, rather than being directly propagated into the final TG-43 parameters. The TG-43 dose-rate constant (Λ) was further validated through direct measurement using both EBT3 film and an XR multidetector, a detector optimized for low-energy photon dosimetry with reduced energy dependence. In contrast, the radial dose function and anisotropy function were derived entirely from MC calculations, where statistical and geometric uncertainties were propagated through quadrature summation of tally errors, voxelization effects, and medium correction factors, in line with TG-138 guidance.

One of the main limitations of the mXT involves inter-device dosimetric variability, which may arise from the CNT coating process during manufacturing. This variation necessitates acceptance testing and individualized dosimetric verification for each device prior to clinical implementation. Additionally, energy spectrum consistency and potential beam hardening effects remain technical challenges that require further optimization. Furthermore, the physical size and limited photon energy output (≤70 kVp) of the mXT restrict its application to superficial or shallow-depth targets. It is not suitable for deep-seated tumors typically treated with high-energy sources such as Ir-192 or Co-60. However, it may be clinically appropriate for HDR treatments using rigid applicators—such as vaginal cylinders—where shallow dose delivery is sufficient and potentially advantageous for sparing nearby organs-at-risk. In addition, its advantages—including extended lifetime, excellent stability, and high dose-rate output—may outweigh these limitations in selected clinical settings. The mXT’s ability to operate at variable voltages and currents also highlights its potential applicability in intensity-modulated brachytherapy.

From a developmental perspective, the mXT inherently involves manufacturing processes—such as CNT coating on the cathode and vacuum sealing—that can introduce slight inter-device variability in output characteristics. Therefore, before clinical application, individual acceptance testing for each manufactured mXT would be essential to ensure dosimetric consistency. The TG-43 dosimetric parameter derivation process proposed in this study could serve as a standardized acceptance testing methodology for such devices. Furthermore, the derived dosimetric parameters can not only be used for direct clinical commissioning but also serve as a basis for constructing a more flexible dose calculation engine that allows parameter adjustments through modeling rather than relying solely on a fixed MC dataset. In a broader clinical context, the methodology presented here could improve treatment reproducibility by providing a validated and traceable framework for verifying mXT performance over time. However, for routine clinical adoption, the current dosimetric workflow would need to be streamlined and simplified, which warrants further research. Future studies should therefore focus on developing a more efficient parameter-based modeling approach and integrating it into clinical QA workflows to support reliable and reproducible HDR brachytherapy with mXT sources.

## 5 Conclusion

In this study, we successfully determined the dosimetric parameters for the mXT, and the determined dosimetric parameters confirm the feasibility of mXT for clinical HDR brachytherapy. These results demonstrate that the vacuum-sealed mXT, with its measured and simulated dosimetric characteristics, meets dosimetric acceptability criteria for use in HDR brachytherapy. Further integration into clinical workflows or treatment planning systems may be warranted.

## Data Availability

The datasets presented in this study can be found in online repositories. The names of the repository/repositories and accession number(s) can be found in the article/supplementary material.
